# Beyond the Intensive Care Unit (ICU): Countywide Impact of Universal ICU *Staphylococcus aureus* Decolonization

**DOI:** 10.1093/aje/kww008

**Published:** 2016-02-11

**Authors:** Bruce Y. Lee, Sarah M. Bartsch, Kim F. Wong, James A. McKinnell, Eric Cui, Chenghua Cao, Diane S. Kim, Loren G. Miller, Susan S. Huang

**Keywords:** decolonization, hospitals, intensive care unit, MRSA, MSSA, nursing homes

## Abstract

A recent trial showed that universal decolonization in adult intensive care units (ICUs) resulted in greater reductions in all bloodstream infections and clinical isolates of methicillin-resistant *Staphylococcus aureus* (MRSA) than either targeted decolonization or screening and isolation. Since regional health-care facilities are highly interconnected through patient-sharing, focusing on individual ICUs may miss the broader impact of decolonization. Using our Regional Healthcare Ecosystem Analyst simulation model of all health-care facilities in Orange County, California, we evaluated the impact of chlorhexidine baths and mupirocin on all ICU admissions when universal decolonization was implemented for 25%, 50%, 75%, and 100% of ICU beds countywide (compared with screening and contact precautions). Direct benefits were substantial in ICUs implementing decolonization (a median 60% relative reduction in MRSA prevalence). When 100% of countywide ICU beds were decolonized, there were spillover effects in general wards, long-term acute-care facilities, and nursing homes resulting in median 8.0%, 3.0%, and 1.9% relative MRSA reductions at 1 year, respectively. MRSA prevalence decreased by a relative 3.2% countywide, with similar effects for methicillin-susceptible *S. aureus*. We showed that a large proportion of decolonization's benefits are missed when accounting only for ICU impact. Approximately 70% of the countywide cases of MRSA carriage averted after 1 year of universal ICU decolonization were outside the ICU.

Methicillin-resistant *Staphylococcus aureus* (MRSA) is considered a serious public health threat by the Centers for Disease Control and Prevention (1). MRSA infection results in substantial morbidity and mortality and can lead to increases in hospital costs and lengths of stay (2, 3). In 2011, an estimated 14,156 hospital-onset invasive MRSA infections occurred in the United States, 26% of which were in intensive care units (ICUs) (4). ICU patients are at risk for MRSA infections, which are associated with worse clinical outcomes (3, 5, 6). Additionally, ICUs serve as a reservoir and can be a means of new acquisition of MRSA among previously uncolonized or uninfected persons (7). Because asymptomatic colonization often precedes infection (8, 9), prevention strategies to reduce infection have included screening admitted patients for MRSA and placing them in contact precautions (single room, use of a gown and gloves for all contact) to prevent transmission, increased environmental cleaning, and decolonization using antiseptic soaps and nasal ointments to remove MRSA from the body.

A recent large trial (the REDUCE MRSA Trial) showed that universal decolonization of all patients without MRSA screening in adult ICUs resulted in a significantly greater reduction in MRSA clinical isolates than either targeted decolonization or screening and isolation (10). Universal ICU decolonization significantly reduced MRSA-positive clinical cultures by 37% and all-cause bloodstream infection by 44%. Additionally, ICUs needed to decolonize 181 patients to prevent 1 MRSA-positive clinical culture and 99 patients to prevent 1 bloodstream infection from any pathogen (10).

Since health-care facilities in a region are highly interconnected through patient-sharing, focusing on individual facilities may miss the potential broader impact of decolonization. Previous work in Orange County, California, has demonstrated the extent to which patients move among health-care facilities (acute-care, long-term acute-care, and nursing homes) via both direct transfers and readmissions after discharge (11, 12). Patients can thus carry pathogens such as MRSA from one facility to another (13–15). Therefore, decolonization of MRSA carriers in an ICU in a hospital could potentially reduce transmission in the rest of the hospital as well as to other health-care facilities to which those carriers would later transfer. To determine the countywide impact of MRSA decolonization in ICUs on MRSA and methicillin-susceptible *S. aureus* (MSSA) prevalence and numbers of carriers, we utilized a computational simulation model of all inpatient health-care facilities and the community in Orange County.

## METHODS

### Model and data sources

Using our custom-designed software, the Regional Healthcare Ecosystem Analyst (RHEA) (13, 16), we expanded our previous work to assess the impact of ICU decolonization with regard to MRSA. In brief, the RHEA-generated agent-based model represents all adult acute-care facilities and nursing homes in Orange County (102 health-care facilities in total; 28 hospitals, including 5 long-term acute-care facilities (LTACs), and 74 nursing homes) (13–19). Orange County is the sixth largest county in the United States, with a population of 3.1 million. Patient movement between and among the various types of health-care facilities and the community has been previously described (15, 16, 19).

The model used 2011–2012 patient-level data for all adult inpatient admissions from the 102 facilities (20, 21) and included parameters derived from extensive data sources in Orange County (which have been previously described (17, 22)). Briefly, we utilized facility-specific line-item admission and discharge data to establish our model, including hospital admission volume to general wards and ICUs, facility length-of-stay distributions, proportions of patients readmitted by facility, and facility-specific distributions of locations and times to readmission among those readmitted. Knowledge of transfer distributions and locations included transfers that occurred directly between facilities and those that occurred with an intervening stay at home or elsewhere (e.g., nursing homes). Our model assumed that upon transfer to an acute-care hospital, 50% of LTAC patients and 20% of nursing home residents (23) would be admitted to the ICU, representing those patients who require mechanical respiratory ventilation or other forms of intensive care.

Patients in the model could be *S. aureus* carriers or noncarriers, with carriers harboring either MRSA or MSSA. In each health-care facility, the total prevalence of *S. aureus* colonization was set to 30% (9) at day 0. The MRSA prevalence in each facility was based on Orange County hospital- and nursing home-specific prevalence data (24–27), while the remaining portion of *S. aureus* carriers in each facility were colonized with MSSA (i.e., for each hospital, MSSA prevalence = 30% −MRSA prevalence). We assumed a certain influx of MRSA from the community (e.g., admissions from the community). For hospitals, this influx was optimized such that the model targeted the facility-specific point prevalence data; for nursing homes, it was set to 10% (27). In our model, the facility-specific length-of-stay distributions for MRSA-positive patients were longer than the distributions for MRSA-negative patients (an average of 5.5 days longer countywide), based on published facility-specific data (20).

MRSA transmission in RHEA has been described elsewhere (13, 15, 16, 18, 19). Briefly, transmission occurred in each ward in each facility on each day and depended on the ward transmission coefficient (β) and the number of susceptible and infectious individuals in that ward. We parameterized facility-specific and ward-specific transmission coefficients to provide a target incidence of 0.01, 0.03, and 0.02 cases per number of susceptible annual ward admissions for general wards, ICUs, and LTACs, respectively (13), and to provide the target prevalence (based on Orange County data (24)) for nursing homes. Various interventions (described below) attenuated transmission by their compliance and/or efficacy. Additionally, MRSA carriage was deemed to be persistent for one-third of carriers (9), while the remaining two-thirds experienced a linear spontaneous loss (25% over 274 days after initial colonization (28)). All patients, regardless of colonization status (i.e., colonized and uncolonized patients), had a risk of developing MRSA or MSSA infection (Table 1). Infection was assumed to last for 10 days and to increase a patient's length of stay by 4 days (3, 29).

**Table 1.** Key Input Parameters, Values, and Sources Used in the RHEA Model to Simulate the Impact of Hospital Decolonization Procedures on *Staphylococcus aureus* Carriage in Orange County, California

Parameter	No.	Median (Range)^a^	Mean (SD)^b^	%	Source (Reference No.)
All *S. aureus* carriage (in acute-care facilities and nursing homes)^c^				30.0	9
MRSA prevalence at baseline, %					
In acute-care facilities		0.034 (0.011–0.185)			25, 26
In nursing homes		0.259 (0.0–0.52)			24, 27
MRSA incidence^d^					
In general wards	0.01				
In ICUs	0.03				
In LTACs	0.02				
In nursing homes^e^			0.20 (0.12)		
MRSA transmission coefficient^e^					
In general wards			0.001757 (0.000728)		
In ICUs			0.007280 (0.007693)		
In LTACs			0.001216 (0.000993)		
In nursing homes			0.000083 (0.000075)		
Persistent MRSA carriers				33	9
Spontaneous loss for MRSA (over 274 days)				25	28
Infection risk (all body sites) per 1,000 patient days^f^					
MRSA infection if MRSA carrier					
In ICUs	12.46				10, 43, 44, 52
In non-ICUs	5.96				10, 44, 52, 53
In nursing homes	0.75				54–57
MRSA infection if non-MRSA carrier^g^					
In ICUs	0.73				10, 43, 52
In non-ICUs	0.24				10, 52, 53
In nursing homes	0.15				55–57
MSSA infection if MRSA carrier					
In ICUs	4.04				10, 43
In non-ICUs	1.60				10
In nursing homes	0.25				54
MSSA infection if non-MRSA carrier^g^					
In ICUs	2.19				10, 43
In non-ICUs	0.80				10, 53
In nursing homes	0.25				54, 57
Intervention parameters					
Active surveillance cultures					
Sensitivity				75	30–33
Specificity				97.1	34
Turnaround time, days	2				34
Contact precaution compliance				70	58–62
Decolonization					
Efficacy of chlorhexidine with mupirocin (eradication by day 5)				90	35–39
Relapse after 90 days				20	28, 40
Relapse after 240 days				32	28, 40, 41

Abbreviations: ICU, intensive care unit; LTAC, long term-acute-care facility; MRSA, methicillin-resistant *Staphylococcus aureus*; MSSA, methicillin-susceptible *Staphylococcus aureus*; RHEA, Regional Healthcare Ecosystem Analyst; SD, standard deviation.

^a^ Median (range) across Orange County facilities.

^b^ Mean (SD) across all facilities with that type of ward.

^c^ For MRSA and MSSA at day 0 in the model and maintained for hospitals.

^d^ Number of cases per number of susceptible annual ward admissions.

^e^ Values were derived from the model and were facility- and ward-specific.

^f^ Number of infections resulting from the different carriage state specified.

^g^ Includes non-*S. aureus* carriers and MSSA carriers.

### Interventions

Our model utilized ICU screening for MRSA, contact precautions for MRSA carriers, and decolonization for all ICU patients. Table 2 summarizes the modeled intervention scenarios. For scenarios utilizing screening, each patient was screened upon entering the ICU. Patients in whom the screening test was positive for MRSA were placed under contact precautions. We previously modeled active surveillance cultures with subsequent contact precautions for those patients testing positive (i.e., true and false positives), regardless of true colonization (18). Active surveillance cultures consisted of a nares swab with a sensitivity of 75% (30–33), specificity of 97.1% (34), and turnaround time of 2 days (34). In the current model, only ICU admissions were actively screened for MRSA (consistent with several state laws). Contact precautions were applied to all persons testing positive for MRSA or with a history of MRSA (upon admission to the same hospital). In nursing homes, contact precautions were applied only to persons with clinically apparent MRSA infection (assumed to persist for 10 days). As previously described (18, 19), MRSA transmission was reduced by the effectiveness (combination of compliance and efficacy) of contact precautions, set at 70%.

**Table 2.** Intervention Scenarios for the Impact of Hospital Decolonization Procedures on *Staphylococcus aureus* Carriage in Orange County, California

	Intervention Strategy	
	Active Surveillance and Contact Precautions	Universal ICU Decolonization
ICU patient	Active screening of the nares upon admission, with subsequent contact precautions if positive	Hospitals with participating ICUs: decolonization with daily chlorhexidine baths plus mupirocin for 5 days and contact precautions for known carriers
		Hospitals with nonparticipating ICUs: active surveillance and contact precautions
General ward patient	Contact precautions if known carrier	Contact precautions if known carrier
Nursing home resident	Contact precautions for clinically apparent infections for 10 days	Contact precautions for clinically apparent infections for 10 days

Abbreviation: ICU, intensive care unit.

In our model, universal decolonization was given to all ICU patients upon admission and consisted of daily chlorhexidine baths plus mupirocin for 5 days, consistent with a recent randomized clinical trial (10). The effective success rate of decolonization was 90% after 5 days (35–39). We assumed an equal probability of being decolonized each day between day 1 and day 5. Of those persons successfully decolonized, 20% relapsed after 90 days (28, 40) and 32% relapsed after 240 days (28, 40, 41)—a loss rate that was assumed to be linear over time.

### Experiments and outcomes

Our baseline scenario consisted of active surveillance cultures in ICUs and contact precautions for patients identified as harboring MRSA in any ward. This baseline was compared against ICU decolonization scenarios. To evaluate potential synergistic effects, we analyzed countywide impacts on MRSA and MSSA prevalence when 25%, 50%, 75%, and 100% of ICU beds had a universal decolonization protocol in place. Universal decolonization was implemented by hospital size measured in number of ICU beds, starting with the largest. We thus implemented universal decolonization in the ICUs of 2, 7, 13, and all hospitals (*n* = 23, since the 5 LTACs did not have ICUs), which represented 23%, 51%, 74%, and 100% of ICU beds countywide, respectively (because we did not allow for partial implementation, decolonization was implemented in all ICUs of a hospital). Additional experiments varied contact precaution effectiveness (50%–70%) and decolonization efficacy (75%–90%) when decolonization was implemented for 100% of ICU beds.

We ran 50 simulations for each experiment; each simulation consisted of 1,000 iterations (50,000 total). The model proceeded in 1-day time steps and simulated 9 years after a run-in equilibration period. The impact of decolonization was the difference between scenarios with decolonization and the baseline scenario. Outcomes of interest included the relative changes in MRSA and MSSA prevalence and the number of carriers. By comparing the numbers of carriers across various scenarios, we determined the number of cases of MRSA carriage averted (hereafter called “carriers averted”), or those cases of MRSA carriage that would have occurred had decolonization not been performed.

## RESULTS

### Direct gain in ICUs implementing universal decolonization

**Figure 1.**
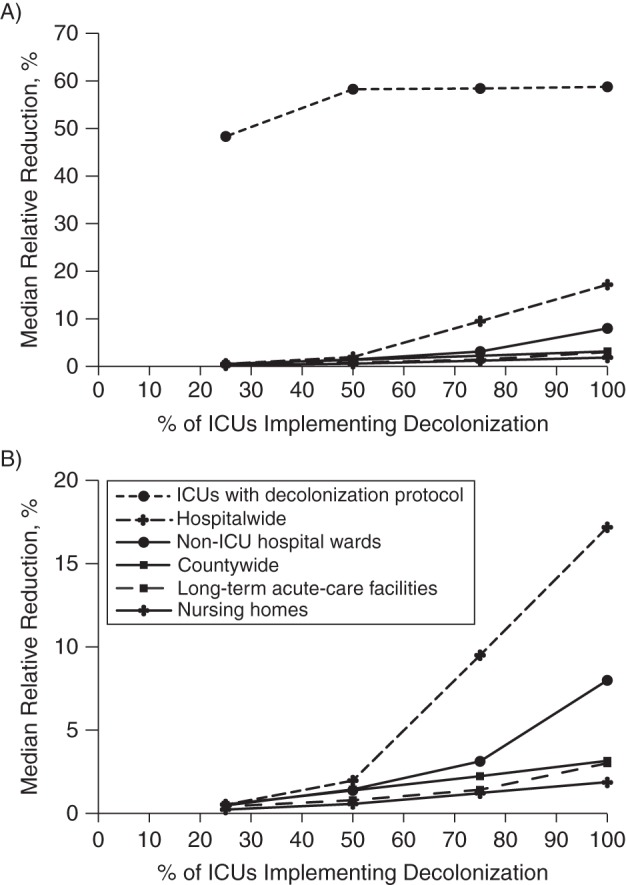
Median relative reduction in the prevalence of methicillin-resistant *Staphylococcus aureus* (MRSA) carriage when increasing the percentage of Orange County, California, intensive care units (ICUs) implementing universal decolonization (90% efficacy) as compared with screening and contact precautions (70% effectiveness). A) Impact in ICUs with decolonization protocols, hospitalwide (all acute-care hospitals), non-ICU wards (general hospital wards), long-term acute-care facilities, and nursing homes. B) Zoom-in of graph shown in part A, excluding ICUs with decolonization protocols.

**Figure 2.**
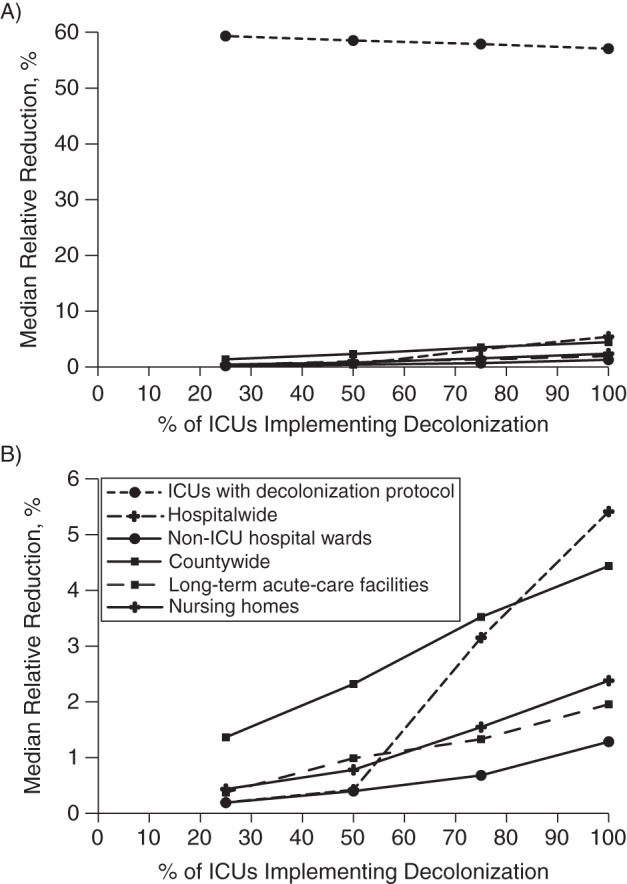
Median relative reduction in the prevalence of methicillin-susceptible *Staphylococcus aureus* (MSSA) carriage when increasing the percentage of Orange County, California, intensive care units (ICUs) implementing universal decolonization (90% efficacy) as compared with screening and contact precautions (70% effectiveness). A) Impact in ICUs with decolonization protocols, hospitalwide (all acute-care hospitals), non-ICU wards (general hospital wards), long-term acute-care facilities, and nursing homes. B) Zoom-in of graph shown in part A, excluding ICUs with decolonization protocols.

Table 3 shows the differences in MRSA prevalence (and 95% confidence intervals) by hospital when implementing decolonization (efficacy 90%) in 25%, 50%, 75%, and 100% of Orange County ICU beds, as compared with no decolonization. Reductions in MRSA prevalence were statistically significant in implementing ICUs. Figure 1 shows the median relative change in MRSA prevalence when comparing decolonization to screening and contact precautions across all decolonizing and nondecolonizing ICUs. Regardless of the number of ICU beds for which decolonization was implemented, ICUs implementing the universal decolonization protocol reduced their MRSA prevalence by approximately half 1 year after implementation. There was a median 48% relative reduction (range, 40%–56%) when decolonization was implemented for 25% of countywide ICU beds and a median 58% relative reduction (range, 35%–70%) when it was implemented for ≥50% of ICU beds. Additional gains continued to accrue but were negligible. For example, hospital A showed a stable 56% relative decrease in MRSA prevalence, even when the number of ICU beds decolonized increased countywide. While decolonization averted 58% of MRSA carriers in ICU wards (15 carriers as compared with 35 carriers with screening), this reduction represented only 28% of MRSA carriers averted countywide (20 of 72 carriers averted countywide were in ICUs) 1 year after decolonizing all ICU patients. Similar reductions were seen for MSSA prevalence (the median relative reduction was 58% (range, 49%–63%), regardless of the number of ICUs implementing decolonization; Figure 2).

**Table 3.** Difference in the Prevalence of Methicillin-Resistant *Staphylococcus aureus* 1 Year After Implementing Universal Decolonization With Chlorhexidine and Mupirocin in Intensive Care Units as Compared with Active Surveillance in Orange County, California, Hospitals (Contact Precaution Effectiveness 70% and Decolonization Efficacy 90%)

Orange County Hospital^a^	No. of Modeled ICU Beds^b^	% of ICUs Implementing Decolonization Countywide							
		25%		50%		75%		100%	
		Difference in MRSA Prevalence	95% CI	Difference in MRSA Prevalence	95% CI	Difference in MRSA Prevalence	95% CI	Difference in MRSA Prevalence	95% CI
A	60	4.8	4.7, 4.8	4.8	4.7, 4.8	4.8	4.7, 4.8	4.8	4.8, 4.9
B	48	2.5	2.4, 2.5	2.5	2.5, 2.6	2.5	2.5, 2.6	2.5	2.5, 2.6
C	36	0.0	0.0, 0.1	6.7	6.7, 6.8	6.7	6.6, 6.8	6.7	6.7, 6.8
D	24	0.1	0.0, 0.2	6.4	6.3, 6.5	6.4	6.3, 6.4	6.4	6.3, 6.5
E	24	0.1	0.0, 0.3	8.5	8.4, 8.6	8.5	8.4, 8.6	8.5	8.4, 8.6
F	24	0.0	−0.1, 0.1	8.1	8.1, 8.2	8.2	8.2, 8.3	8.2	8.2, 8.3
G	24	0.0	−0.1, 0.1	2.9	2.8, 2.9	2.8	2.8, 2.9	2.8	2.8, 2.9
H	24	0.0	−0.1, 0.1	0.0	−0.1, 0.1	7.8	7.7, 7.9	7.8	7.7, 7.9
I	24	0.1	0.0, 0.2	0.1	0.0, 0.2	3.1	3.1, 3.2	3.2	3.1, 3.3
J	24	0.0	−0.1, 0.1	0.1	0.0, 0.2	2.0	1.9, 2.0	2.0	1.9, 2.1
K	12	0.0	−0.1, 0.1	0.1	0.0, 0.2	8.4	8.3, 8.5	8.4	8.3, 8.5
L	12	0.0	−0.2, 0.1	0.1	0.0, 0.3	9.9	9.8, 10.0	9.9	9.8, 10.0
M	12	0.0	−0.1, 0.2	0.2	0.0, 0.3	10.3	10.2, 10.4	10.2	10.1, 10.4
N	12	0.1	−0.1, 0.3	0.2	0.0, 0.4	0.2	0.1, 0.4	11.1	11.0, 11.2
O	12	−0.1	−0.2, 0.0	0.0	−0.2, 0.1	0.0	−0.1, 0.2	11.6	11.5, 11.7
P	12	0.2	0.0, 0.3	0.2	0.1, 0.4	0.2	0.0, 0.3	2.3	2.2, 2.5
Q	12	0.0	−0.2, 0.2	0.0	−0.2, 0.3	0.2	−0.1, 0.4	11.8	11.6, 12.0
R	12	0.1	−0.1, 0.2	0.1	−0.1, 0.3	0.3	0.1, 0.4	3.6	3.5, 3.7
S	12	0.0	−0.2, 0.2	0.0	−0.2, 0.3	0.1	−0.1, 0.4	11.9	11.7, 12.1
T	12	0.0	−0.1, 0.2	0.2	0.01, 0.3	0.4	0.2, 0.5	5.9	5.8, 6.0
U	12	−0.1	−0.3, 0.2	0.1	−0.2, 0.4	0.1	−0.2, 0.4	5.7	5.4, 6.0
V	12	0.1	−0.1, 0.2	0.1	0.0, 0.3	0.1	−0.1, 0.2	2.9	2.7, 3.0
W	12	0.2	−0.1, 0.5	0.1	−0.2, 0.4	0.1	−0.2, 0.5	7.6	7.3, 7.8

Abbreviations: CI, confidence interval; ICU, intensive care unit; MRSA, methicillin-resistant *Staphylococcus aureus*.

^a^ Hospitals are rank-ordered by total number of ICU beds, starting with the largest. Decolonization in hospitals A and B represents 25% of countywide ICU beds undergoing decolonization; decolonization in hospitals A–G represents 50% of countywide ICU beds undergoing decolonization; decolonization in hospitals A–M represents 75% of countywide ICU beds undergoing decolonization; and decolonization in hospitals A–W represents 100% of countywide ICU beds undergoing decolonization.

^b^ Each ICU ward had 12 beds.

Results were similar for decolonization efficacy of 75%. Trends were similar over time, with median relative reductions in MRSA and MSSA prevalence of 47.5% (range, 25.6%–57.7%) and 47.9% (range, 41.1%–52.1%), respectively, in ICUs with decolonization procedures when decolonizing all ICU patients. Decolonization resulted in larger gains with lower contact precaution effectiveness (50%), with median relative reductions in MRSA and MSSA prevalence of 60% and 57%, respectively, when decolonizing all ICUs.

### Indirect gain in general wards and hospitalwide for hospitals implementing decolonization in ICUs

After 1 year, when decolonization was implemented for all countywide ICU beds, general wards in hospitals that implemented ICU decolonization saw a median 8.0% relative reduction in MRSA prevalence (range, 0.7%–15.7%); 3 of 28 hospitals had a relative decrease of ≥10%. These benefits increased slightly over time (9.9% median relative reduction after 3 years; range, 0.8%–17.0%). When all Orange County ICU beds were decolonized, 47% of the countywide cases of MRSA carriage were averted after 1 year of decolonization in hospitals with decolonization procedures, with 19% of the total reduction occurring in non-ICU wards. After 6 years, 31% and 15% of countywide MRSA carriers averted were in hospitals with decolonization procedures and non-ICU wards, respectively. Results were similar with a lower decolonization efficacy (75%); general wards in hospitals with decolonization procedures achieved a median 5.9% relative reduction for a 13.6% relative reduction hospitalwide after 1 year when decolonizing all ICU patients. Decolonization resulted in larger reductions when reducing the effectiveness of contact precautions (a median 17.8% (range, 2.6%–24.5%) relative reduction hospitalwide when decolonizing all ICU patients).

Figure 2 shows the median relative change in MSSA prevalence. The indirect MSSA benefits garnered by hospitals implementing decolonization protocols were less than those for MRSA. The median relative reduction in prevalence was 1.3% (range, 0.4%–9.9%) in the general wards for which their ICU counterparts were decolonizing (100% of ICU beds countywide decolonized). Hospitalwide (both ICU and general wards), ICU decolonization led to a median 5.43% (range, 2.9%–17.6%) relative reduction in MSSA prevalence when all ICUs were decolonized. Again, benefits were largely reaped within 1 year of implementation, and results were similar with a lower decolonization efficacy and reduced contact precaution effectiveness.

### Indirect gains in other hospitals, LTACs, and nursing homes and countywide

Table 3 and Figure 1 also show the indirect benefits to ICUs not implementing decolonization. Little effect was seen in the ICUs in hospitals not implementing decolonization (a median 2.6% (range, 0.9%–5.3%) relative reduction in MRSA prevalence when 75% of Orange County ICU beds were decolonized). However, some reductions in MRSA prevalence, although small, were statistically significant. Decolonization led to significant reductions in MRSA prevalence in ICUs of 2 of the 16 hospitals not implementing decolonization when 50% of countywide ICU beds underwent decolonization, and 3 of 10 when 75% underwent decolonization (Table 3). Acute-care hospitals not implementing ICU decolonization saw median 0.4%, 1.2%, and 2.3% relative reductions in their total MRSA prevalence when 25%, 50%, and 75% of countywide ICU beds were decolonized, respectively.

Figure 1 shows modest indirect benefits in other Orange County health-care facilities, LTACs, and nursing homes. The reduction in MRSA prevalence seen in LTACs was small (the maximum for any LTAC was a 3.2% reduction) but linearly affected by the number of ICUs implementing decolonization (Figure 1). Although the change was small (maximum 4.5% reduction), a vast majority of nursing homes (93%) showed a reduction in MRSA prevalence when 100% of ICUs implemented decolonization, ranging from 0.1% to 4.5%. Overall, the median countywide MRSA prevalence in all health-care facilities decreased by a relative 3.2% when 100% of ICU beds underwent decolonization. Countywide MRSA reductions increased over time and were statistically significant. At 1 year, decolonization resulted in a 0.66 absolute difference in countywide prevalence (95% confidence interval (CI): 0.65, 0.67); at 6 years, the difference in prevalence was 1.14 (95% CI: 1.13, 1.15). These differences, although small, were statistically significant even when only 25% of countywide ICU beds were decolonized (at 1 year, the difference was 0.11; 95% CI: 0.10, 0.12). Changes in the countywide prevalence were largely driven by the changes in the ICUs themselves.

As more countywide ICUs implemented decolonization, the number of MRSA carriers in hospitals not implementing ICU decolonization, LTACs, and nursing homes decreased. After 1 year, approximately 53% of countywide cases of MRSA carriage averted were outside of hospitals with decolonized ICU beds (regardless of the number of Orange County ICU beds decolonized). These benefits in other facilities accrued over time; the proportion of averted cases in other hospitals and facilities increased to approximately 70%, regardless of the number of ICU beds decolonized, with a majority of the benefits accruing in nursing homes. When 100% of ICUs were decolonized, 53% of countywide MRSA carriers averted after 1 year were in non–acute-care facilities (1.5% in LTACs and 51.5% in nursing homes); this figure increased to 69% after 6 years.

Decreasing decolonization efficacy (75%) had little impact on the relative reduction in LTACs (2.2%) and nursing homes (1.5%). Countywide, a 2.5% relative reduction was achieved after 1 year of decolonizing all ICU patients. Decreasing the effectiveness of contact precautions increased the benefits of decolonization. LTACs and nursing homes garnered median 3.3% and 2.1% relative reductions, respectively, and countywide a median 3.5% relative reduction in MRSA prevalence was achieved after 1 year of decolonizing all ICU patients.

For MSSA (Figure 2), the effect of universal ICU decolonization on other hospitals was minimal, resulting in a median 0.5% relative decrease (range, no effect to 1.2%) 1 year after decolonization protocols were implemented for 75% of ICU beds countywide. Nursing homes and LTACs garnered similar reductions with MSSA as they did with MRSA. LTACs experienced a median 2.0% relative decrease (range, 0.1%–3.7%), while nursing homes experienced a median 2.4% relative decrease (range, no effect to 5.0%) when 100% of Orange County ICUs implemented decolonization (1 year after). Overall, the county saw 1.4%, 2.3%, 3.5%, and 4.4% relative reductions in its MSSA prevalence after 1 year when universal decolonization measures were implemented in 25%, 50%, 75%, and 100% of countywide ICU beds, respectively. Trends were similar for reduced decolonization efficacy and contact precaution effectiveness.

## DISCUSSION

When evaluating *S. aureus* carriage, universal decolonization in ICUs produced direct and rapid reductions in MRSA and MSSA prevalence, halving overall ICU levels of both within a year. Clinical trial findings suggest that this strategy significantly reduces transmission and health-care-associated infection risk (10). After 1 year, MRSA prevalence continued to drop approximately 0.5% per year. However, reductions in ICUs implementing decolonization only represented approximately 30% of countywide MRSA carriers averted 1 year after implementing decolonization; the remaining averted carriers were in nondecolonizing hospitals, LTACs, and nursing homes. This decreased to 17% of averted countywide MRSA carriers 6 years after implementation as continued reductions in MRSA prevalence and transmission accrued in non-ICU settings. Thus, a large proportion of decolonization's benefits are missed when measuring only ICU MRSA prevalence and not considering secondary benefits derived from reduced numbers of carriers exiting ICUs and transferring to other wards and facilities. While the indirect effects of ICU decolonization were modest in any given setting, they accrued to a large number of carriers, since the numbers of patients in non-ICU settings are far greater than those in ICU settings. Universal ICU decolonization led to significant reductions in MRSA prevalence countywide (even when only 25% of ICUs were decolonized) but did not completely eradicate MRSA in any hospital. While the reduction in MSSA prevalence was similar to that of MRSA in decolonizing ICUs, reductions were not similar in other wards as decolonization had more impact on MRSA patients. Specifically, MRSA patients experience longer stays that allow for completion of decolonization. MRSA patients also tend to remain in the hospital network longer after discharge compared with MSSA patients.

As decolonization in ICUs moves toward becoming the standard of care (42), our work provides an example of the greater direct and indirect impact this process may have on health-care facilities in a region. While a majority of benefits occurred in the ICUs implementing decolonization, some effects spilled over to other wards in the same facility and to other facilities. These indirect benefits were generally modest, suggesting that ICUs tend to be relatively isolated and that MRSA control measures implemented exclusively in ICUs may have a small impact on the total MRSA prevalence. Prevention and control measures implemented in ICUs are not far-reaching; therefore, implementation of such measures in other areas (e.g., general wards, nursing homes) may garner larger benefits and yield a larger impact on the overall prevalence of *S. aureus.*

For this study, our model focused on *S. aureus* carriage. While MRSA carriage is not a necessary precursor to MRSA infection (43), MRSA carriage is a well-known precursor of MRSA infection (8, 44, 45), with infection rates varying from 5.1% in 6 months to 33.1% within 1 year. Human immunity does not readily arise from carriage; in fact, carriage strains seem to be genetically the same as strains resulting in infection (8, 46). These findings, along with robust evidence from clinical trials that show decreases in infection rate from decolonization of 37%, support the value of decolonization in preventing infection (10, 47).

Cost and cost-effectiveness models evaluating ICU universal decolonization, as compared with targeted decolonization and screening and contact precautions alone, show high savings (7, 48, 49). These savings could be even greater if they accounted for the benefits garnered by other wards and facilities. It will remain to be seen whether antiseptic resistance to chlorhexidine and antibiotic resistance to mupirocin emerges over time and impacts cost and benefit estimates (50).

All models, by nature, are simplifications of real life (51). Our model assumed homogeneous mixing within wards and nursing homes. We did not model specific types of disease (i.e., infection), as our intent was to evaluate regional synergistic changes in *S. aureus* prevalence over time. While the impact to infection is already well described in the clinical trials (10, 47), our future work aims to incorporate more details on infection. We did not include pediatric patients and hospitals or hospitals outside Orange County, although 90% of Orange County hospital patients stay within the county for care (17). Our model does not account for potential antimicrobial resistance to mupirocin and chlorhexidine. Additionally, we did not model potential adverse effects of contact precautions or decolonization or evaluate the impact of contact precautions on other antibiotic-resistant organisms. Although Orange County's health-care facilities vary in size and type and serve a diverse population, our findings may not be representative of all counties or regions.

### Conclusion

In our simulations, the impact of universal decolonization in ICUs on MRSA prevalence was substantial in the ICUs implementing decolonization; however, more than half of the benefit (approximately 70%) in averted MRSA carriers was seen downstream in non-ICU settings (i.e., general wards, nondecolonizing hospitals, LTACs, and nursing homes). Nevertheless, while the total numbers of averted carriers countywide were high over time, the facility-specific indirect effects on MRSA prevalence were modest. Our findings suggest that universal ICU decolonization will have some spillover effect and synergy but will not substantially contribute to a countywide MRSA eradication program, thereby warranting the broader use of decolonization beyond the ICU or other control measures. ICU decolonization should be coordinated across hospitals in a region, but coordinated ICU efforts alone are not enough to eradicate MRSA in a region.
